# Digital genetic counseling services for cascade cardiogenetic testing

**DOI:** 10.1002/jgc4.70208

**Published:** 2026-04-29

**Authors:** Marlies N. van Lingen, Sietske A. L. van Till, Noor A. A. Giesbertz, Tessa C. Beinema, Margreet G. E. M. Ausems, Randy Klaassen, Martina C. Cornel, Lieke M. van den Heuvel, J. Peter van Tintelen

**Affiliations:** ^1^ Department of Genetics University Medical Center Utrecht Utrecht The Netherlands; ^2^ Department of Medical Ethics, Philosophy and History of Medicine Erasmus MC, University Medical Centre Rotterdam Rotterdam The Netherlands; ^3^ Department of Clinical Genetics Antoni van Leeuwenhoek Netherlands Cancer Institute Amsterdam The Netherlands; ^4^ Department of Human Media Interaction University of Twente Enschede The Netherlands; ^5^ Department of Human Genetics, Amsterdam University Medical Center Vrije Universiteit Amsterdam Amsterdam The Netherlands; ^6^ Public Health Research Institute Amsterdam The Netherlands; ^7^ Netherlands Heart Institute Utrecht The Netherlands

**Keywords:** cardiogenetics, cascade testing, digital tools, genetic counseling, user perspective

## Abstract

Digital interventions are potentially promising to improve accessibility and efficiency of genetic counseling services. However, current literature on user perspectives toward digital tools for cascade testing is limited. Therefore, this focus‐group study aimed to gain insights into the attitude and perspectives of probands, at‐risk relatives (ARR), and genetic healthcare professionals (HCP) toward digital innovations for assistance with both pretest and posttest counseling and cascade genetic testing in cardiogenetics. We conducted seven online focus groups, which were transcribed and thematically analyzed. In total, 37 individuals participated (10 probands, 11 ARR and 16 HCP). Thematic analysis of focus group transcripts showed a first theme of (1) acceptability of digital tools. Other identified themes were defined as “domains,” where digital tools impact traditional, in‐person clinical genetic care, being (2) family communication, (3) decision‐making, (4) care relations, and (5) the genetic care system. Participants expressed a predominantly positive attitude toward the digitization of (parts of) the predictive genetic counseling journey in cardiogenetics under the condition that access to human contact is preserved. In the clinical setting of predictive counseling, efforts should be made to ensure access to genetic services for all ARR and to protect in‐person involvement of HCP.

**What is known about this topic**

Digital tools to support genetic counseling are promising for improving the efficiency and accessibility of genetic care. However, little is known about the perspectives of patients, at‐risk relatives, and genetic healthcare professionals on digital technologies supporting cascade genetic counseling and testing.

**What this paper adds to the topic**

Participants expressed a predominantly positive attitude toward the digitization of aspects of predictive genetic counseling in cardiogenetics, provided that meaningful access to human interaction is safeguarded. In clinical practice, efforts should focus on ensuring equitable access to genetic services for all at‐risk relatives.

## 1 INTRODUCTION

The importance of cascade genetic testing for monogenic treatable inheritable diseases is undisputed (Christiaans et al., 2008; Godino et al., 2016; Srinivasan et al., 2020). After identification of the disease‐causing variant in a proband, cascade screening in at‐risk relatives (ARR) is recommended (Lindor et al., 2006; Theilade et al., 2013). In the Netherlands, the family‐mediated approach is adopted, where probands inform their ARR about an inherited disease and genetic testing options, assisted by a family letter from their genetic healthcare professional (HCP) (Heuvel et al., 2019). After a referral by their GP, an ARR typically receives a video, telephone, or in‐person consultation, depending on their preference. Cascade genetic testing, followed by clinical follow‐up and treatment when indicated, is of utmost importance as the process has been proven to decrease morbidity and mortality in relatives at risk of inheritable cardiac and oncological conditions (Theilade et al., 2013). However, the uptake of genetic counseling and/or predictive genetic testing for monogenic treatable diseases ranges between 40% and 60% in cardiogenetics (Burns et al., 2016; Christiaans et al., 2008; Cirino et al., 2022; Shah et al., 2019; Van Den Heuvel et al., 2020) and oncogenetics (Menko et al., 2020, 2023; Sanz et al., 2010).

Several barriers have been identified that hamper probands from informing their ARR or that prevent ARR from obtaining genetic counseling, including family‐related (Campbell et al., 2017; Ho et al., 2022; Srinivasan et al., 2020; Whyte et al., 2016), knowledge (Campbell et al., 2017; Ho et al., 2022) and practical barriers, including financial barriers, limited access to genetic services (Srinivasan et al., 2020), or fear of genetic discrimination by insurers (Kahn et al., 2023). Digital interventions have been identified as potentially promising to improve the accessibility and efficiency of genetic counseling services (Bombard et al., 2022; Lee et al., 2023). Existing digital genetic counseling tools include conversational agents (CA, i.e., chatbots or virtual assistants) (Schmidlen et al., 2022), decision aids (Christian et al., 2021; Shannon et al., 2024; Yee et al., 2014), family outreach tools (Pande et al., 2022), and full pre‐ (Torr et al., 2022) and posttest counseling platforms (Shickh et al., 2022). Families confronted with inheritable cardiac conditions are particularly relevant as a target population for digital intervention for several reasons. First, inherited cardiac conditions consist of mostly autosomal dominant diseases with treatment or preventative options (Arbelo et al., 2023). Both contribute to the actionability of cardiogenetic conditions. Second, probands and ARRs follow a well‐defined genetic care journey (Arbelo et al., 2023). Third, the patient load is substantial and growing (Arbelo et al., 2023). These factors highlight the need for accessible, available, and efficient cascade genetic counseling services for cardiogenetic conditions. Digital interventions in genetic care could save time and workload and enhance the cascade testing process while increasing accessibility (van Lingen et al., 2025). Simultaneously, development and implementation of digital tools for predictive genetic counseling raises ethical and practical questions, including questions related to equitable access, clinical validity, and utility, and involving end‐users during the design (Bombard et al., 2022; van Lingen et al., 2025). It is clear that inclusion of the perspectives of probands, ARR, and HCP toward the development of digital tools are crucial for responsible integration of technologies into genetic care (van Lingen et al., 2025). However, studies on user perspectives toward digital tools for cascade testing are limited, except for studies on attitudes toward chatbots from clinically affected patients (Schmidlen et al., 2019) and ARRs (Siglen et al., 2023).

A previous focus‐group study conducted with 29 US patients receiving clinically actionable variants showed that most participants were willing to use a follow‐up and cascade chatbot while several were willing to share genetic results with ARR via a chatbot (Schmidlen et al., 2019). Still, participants expressed privacy and usability concerns in family communication (Schmidlen et al., 2019). However, it remains unclear how patients with diverse backgrounds or HCP view CA or other digital technologies for family communication and cascade testing. Therefore, this focus‐group study aims to gain insights into attitudes and perspectives of probands, ARR, and HCP toward digital counseling tools to assist family communication and both pre‐ and posttest counseling and cascade genetic testing of inherited cardiac diseases. These insights could benefit sound development of digital tools for cascade genetic testing in line with the needs and values of these families and their caregivers.

## 2 MATERIALS AND METHODS

### 2.1 Design

A qualitative focus‐group design was chosen to gain insights into the attitude and perspectives of those involved and to enhance discussion and formation of new ideas on this novel topic. We adopted a postpositivist paradigm for this study (Wainstein et al., 2023) and used coding reliability thematic analysis as a method to inductively analyze the focus group transcripts (Braun & Clarke, 2006, 2019). The consolidated criteria for reporting qualitative research (COREQ) were used to report methods and results, see Appendix S1 (Tong et al., 2007). The study protocol was exempted from approval by the Medical Ethical Committee NedMec because the Act of Medical Research Involving Human Subjects (WMO) was not applicable (research proposal no. 21‐474/C). We obtained written and verbal informed consent from all participants in the study.

### 2.2 Participants and procedure

Participants were divided into three groups: probands, ARR, and genetic HCP (i.e., clinical geneticists (in training), genetic counselors, genetic social workers, or genetic laboratory specialists). Probands and ARR were 18 years or over, and a (likely) pathogenic variant in an established gene underlying the proband's phenotype had to be present. ARR were defined as first‐ or second‐degree family members. Probands and ARR were invited by a letter if they received in‐person genetic counseling between October 2020 and October 2021 at the UMC Utrecht. Eligible probands and ARR were invited by four HCP based on inclusion criteria. Genetic HCP were eligible if they were involved in cardiogenetic cascade testing in three Dutch University Medical Centres (Utrecht, Amsterdam, Groningen). Written informed consent was obtained from all participants. After obtaining informed consent, emails were sent inviting participants to online focus group sessions utilizing Microsoft Teams software.

During each focus group, one researcher (MH) was available to support participants by telephone to address technical questions or problems. After finalizing all focus groups, participating probands and ARR were asked to invite their ARR who did not obtain genetic counseling to participate in this study during a follow‐up focus‐group session.

### 2.3 Data collection

Seven semi‐structured focus groups were organized between November 2021 and February 2022. They were scheduled when five to eight participants were available. Probands, ARR, and HCP were clustered in separate focus‐group sessions. Participants of each subgroup were actively selected based on age, gender, and digital skills in ARR and probands, and profession for HCP. Participant characteristics were collected prior to the focus group during a telephone consultation by the executing researcher (MvL). These included demographic information (i.e., age, sex, and highest completed level of education), information on digital literacy (i.e., ability to perform digital tasks) and health literacy (i.e., ability to read and interpret and health‐related information), data related to the cardiogenetic disease (i.e., disease type, relation to proband, onset of disease, carrier status), and information on the method used to inform ARR (Appendix S2). Education level subgroup classification was based on the Dutch interpretation of the International Standard Classification of Education: Fields of Education and Training 2013 (StatisticsNetherlands, 2021).

The semi‐structured topic list covered general perspectives on the concept of a digital genetic service, the role of digital technology in informing ARR about genetic risk, online information provision and genetic counseling, the role of CA (3 focus groups discussed chatbots and 4 virtual assistants), and considerations regarding decision‐making and genetic testing (Appendix S3). HCP were additionally asked about the process of informing probands and organizing clinical follow‐up with help from a digital tool.

All online focus‐groups were moderated by a trained female researcher (MvL, MD), who had no pre‐existing relationship with the participants. Two observers (alternating: ID, MH, LvH, NG, SvT) assisted the moderator with taking field notes and covering the topic list. Sessions were video‐recorded (MS Teams) and audio‐recorded (Audacity, Version 3.1). Participants received a 10 Euro gift card for an online retailer.

### 2.4 Data analysis

Audio files were transcribed verbatim. Two researchers (MvL, SvT) independently familiarized themselves with the material by reading the transcripts of one focus group of every subgroup (proband/ARR/HCP). These transcripts were then inductively coded using open coding and attribute coding with Nvivo 12 software (Lumivero, Denver, CO, USA). This resulted in a draft codebook, after which intercoder differences in codes and code definitions were resolved by discussion. This predetermined codebook was used to (re)code transcripts of every focus group by the same two researchers, in line with the coding reliability thematic analysis approach (Braun & Clarke, 2019). After open coding of all focus groups, a final codebook was obtained by a cross‐case analysis of the concepts in the original data and the codebook. LvdH critically assessed samples of coded transcripts to promote consistency of the final codebook with the available data. Themes were identified in different rounds, where overlapping codes were clustered into subthemes and iteratively analyzed for their meaning and context within the data. The meaning of subthemes was determined by discussion between MvL and SvT. After identifying distinct subthemes, they were clustered into themes until consensus was reached between MvL, SvT, and LvdH. We reached theoretical saturation of attitudes and perspectives in different groups: only a few new codes were added during final coding of the transcripts and there was consensus about the meaning of the (sub)themes. Illustrating quotes were selected by MvL. Member checking of results was not performed.

## 3 RESULTS

### 3.1 Study participants

Thirty probands, 46 ARR from 37 families (3 ARR were related to a proband), and 30 HCP were invited to participate. No ARR who did not receive genetic counseling responded after being approached by their relatives. Thirteen probands, 13 ARR, and 18 HCP (43%, 28%, 60%, respectively) consented to participate. Three participants in the proband group later declined to participate due to personal circumstances. Four participants (one proband, one ARR, two HCP) were unavailable at the actual focus group meeting. In total, 37 individuals participated in seven focus groups (10 probands, 11 ARR, and 16 HCP). The two proband focus groups each consisted of five participants; the two ARR focus groups included six and five participants respectively, while the focus groups with HCP consisted of five, seven, and four participants, respectively. Median length of the focus groups was 86 min (range 73–104).

Participant characteristics are presented in Table 1. Six out of 11 ARR were first‐degree relatives. Median proband and ARR age was 54 (range 46–68 years) and 61 years (range 43–74 years), respectively. ARR were directly informed by the proband, and all had received a family letter through the proband, written by a genetic HCP. All ARR pursued predictive genetic testing; three ARR (27%) were identified as carriers. Among HCP, clinical geneticist was the most common profession (44%). All HCPs practiced in the field of cardiogenetics, although more than half of the HCPs practiced in other fields as well (e.g., dysmorphology or connective tissue diseases) at the time of focus group discussions. All participants were born in the Netherlands.

**TABLE 1:** Participant characteristics.

Participant characteristics	Probands (*n* = 10)	ARR (*n* = 11)	Genetic HCP (*n* = 16)
Sex			
Female	6 (60%)	7 (63.6%)	10 (62.4%)
Male	4 (40%)	4 (36.3%)	6 (37.5%)
Age, years (median, range)	54 (46–68)	61 (43–74)	44 (31–62)
Phenotype^a^			
ACM	2 (20%)	4 (36.3%)	
DCM	2 (20%)	3 (27.3%)	
HCM	6 (60%)	4 (36.3%)	
Carrier status affected	10 (100%)	3 (27.3%)	
Education level			
Low	4 (40%)	2 (18.2%)	
Medium	1 (10%)	4 (36.3%)	
High	5 (50%)	5 (45.5%)	
Family status			
Single	0	3 (27.3%)	
With partner, no child	2 (20%)	0	
Alone, with child(ren)	0	1 (9.1%)	
With partner and child(ren)	8 (80%)	7 (63.6%)	
Health literacy^b^			
Low	0	0	
Medium	1 (10%)	3 (27.3%)	
High	9 (90%)	8 (72.7%)	
Digital literacy^b^			
Low	1 (10%)	1 (9.1%)	
Medium	0	4 (36.3%)	
High	9 (90%)	6 (54.5%)	
Profession			
Clinical geneticist in training			2 (12.5%)
Genetic counselor			2 (12.5%)
Clinical geneticist			7 (43.8%)
Laboratory specialist			2 (12.5%)
Social worker			3 (18.8%)
Experience in cardiogenetics, years (median, range)			12.0 (1–34)

Abbreviations: ACM, arrhythmogenic cardiomyopathy; ARR, at‐risk relative; DCM, dilated cardiomyopathy; HCM, hypertrophic cardiomyopathy; HCP, healthcare professional.

^a^ In probands, clinical phenotype is reported. In at‐risk relatives, the phenotype of the affected proband in their family is reported.

^b^ Health literacy was defined as medium if participants needed occasional assistance with interpreting and reading health related information from their GP or hospital and low if help was always required. Similarly, digital literacy was understood as needing occasional or constant assistance with digital tasks such as using a computer, visiting a website or using two‐factor authentication, represented by medium or low levels of digital literacy, respectively.

### 3.2 Themes and subthemes

**FIGURE 1:**
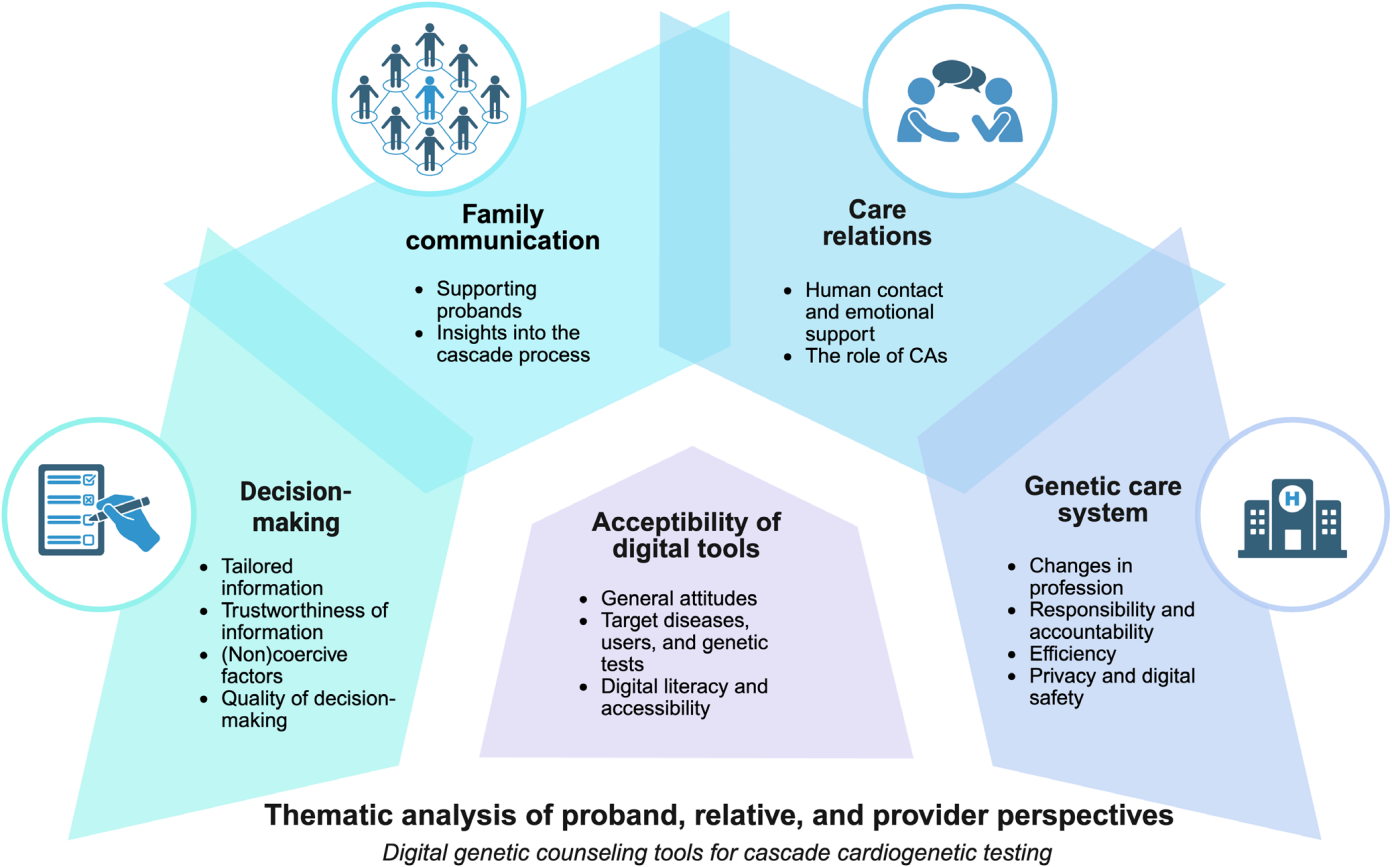
Result of thematic analysis of participant perspectives on digital genetic counseling tools for cascade cardiogenetic testing. Schematic representation of themes (circles) and subthemes derived from thematic analysis of focus groups with probands, at‐risk relatives and healthcare professionals. The central theme represents the acceptability of digital tools and the subthemes that were most relevant to acceptability according to participants. The outer circle represents the domains within regular cascade testing that would be impacted by digital tools, where subthemes capture the main opportunities and challenges identified by participants. CA, conversational agents; e.g. chatbot or virtual assistant.

Five main themes were identified (Figure 1). The first theme involved the general attitude and perspectives of participants toward the acceptability of digital tools in cascade testing. The remaining themes were defined as ‘domains’ in which digital methods impact traditional, in‐person clinical cascade testing: (2) family communication, (3) decision‐making, (4) care relations, and (5) the genetic care system.

### 3.3 Theme 1—Acceptability of digital tools in cascade genetic testing

#### 3.3.1 General attitudes

Participants across all groups expressed a predominantly positive attitude toward digitization of (parts of) family communication and cascade testing. If ARR or probands did not appreciate it for themselves, they envisioned digital pathways to be suitable for younger relatives. Benefits of digital tools included: improved accessibility, with reduced waiting times and the opportunity to relocate parts of genetic counseling to the private environment. However, participants across all groups noted general objections to digitization of pre‐ and/or posttest counseling, including lack of human contact, potential impact on careful deliberation, and the risk of declining care quality.

#### 3.3.2 Target diseases, users, and genetic tests

All participant groups emphasized the importance of considering the target audience in designing and developing digital tools in three ways. First, some HCP stated that the acceptability of using digital predictive counseling tools depends on the characteristics of the targeted genetic condition, including disease severity, penetrance, and availability of treatment options. Clinically milder and treatable conditions, with moderate psychosocial impacts, were considered more suitable (Q1B1).

I think it really depends a lot on what kind of condition it is. And what you can't monitor is the emotional impact and if people start to worry. For example, in my experience with Long QT syndrome, if you tell someone they have a predisposition, people often immediately think, ‘Oh, I might drop dead tomorrow’. (Q1B1; HCP 12)

Many HCP argued that, for counseling cardiogenetic diseases specifically, much information is generic and therefore suitable for digital information transfer. For cascade testing, digital information provision was deemed appropriate for only specific (likely) pathogenic variants. For example, complexities in genetic counseling for variants of unknown significance (VUS) and some likely pathogenic variants might get lost in a digital context. HCP also worried about loss of insights into phenotype‐specific characteristics in ARR and the family pedigree. This might result in a loss of tailored counseling of phenotypical characteristics in ARR that are not yet diagnosed.

Second, participants argued that digital tool design should be adjusted to accommodate the needs of end users. Probands and HCP stated that the variety of ARR's backgrounds complicates design by illustrating why a “one size fits all” approach is not suitable for digital pathways for cascade testing (Q1B2). In their view, interactive modalities such as questionnaires or CA could partly accommodate varying needs.

Because every person is different, of course. One person wants to know every last detail, while another thinks, ‘Yeah, whatever, it's fine with me.’ It's also personal as to how deeply you want to get into it [predictive testing]. And do you want to have a conversation about it or not? (Q1B2; Proband 12)

Some HCP suggested that digital tools should be specifically targeted toward groups that are currently underserved (Q1B3). “We already see a select group that comes to us in the first place. […] But would you also want to reach that other group and see what they want and need?” (Q1B3; HCP 16).

Finally, HCP recommended careful consideration in providing specific genetic tests to counselees in a digital setting. Some considered digital tools more appropriate for predictive genetic testing compared to testing of symptomatic ARR. Few HCP argued that digital tools could contribute to diagnostic pre‐ and posttest counseling of probands, potentially in a mainstreaming setting. Others considered adopting digital tools in a diagnostic setting not suitable, expressing hesitation about such tools to counsel VUS, for example.

#### 3.3.3 Digital literacy and accessibility

Participants across all groups noted a potential lower threshold for ARR to access genetic counseling in a digital setting, as technology was considered widely accessible (Q1C1).

Moderator: If there would be a digital tool where they [your relatives] could find information and possibly apply for a test, do you think this would be suitable for your family? Proband 12: I think they're more likely to do that. That you can look at it first on your computer and always have the option to think: do I want to pursue this or not? But then the first step is taken, without having to make an appointment and visit a cardiac specialist. I think this would be more practical, in the first place. (Q1C1; Proband 12)

Probands thought initiating genetic care would be easier in a digital setting, especially since the Dutch referral process through the GP using a family letter was considered complex. Also, the opportunity to access genetic counseling at a time and place convenient for the counselee was mentioned (Q1C2).

Well, you can do it [using a digital tool] in the evening. You don't have to take off from work to go to the hospital. Although that is an assumption, I don't know if that will happen in reality, but it could be. (Q1C2; HCP 2)

Finally, one HCP noted that digital tools for cascade testing could potentially reach ARR who are currently not receiving genetic counseling as they are reluctant to speak with a HCP or have a digital mindset. However, most HCP argued that digital tools might disproportionally benefit already privileged counselees. They argued that families with strong digital and literacy skills, currently already overrepresented in Dutch genetics clinics, might get even greater access to genetic services. Some HCP stated that this may not necessarily be problematic, as shorter waiting lists create more opportunities for in‐person counseling of less privileged counselees (Q1C3).

I think on the one hand, it is a really nice idea [digital tools] and a way to speed up the diagnostic process [of predictive testing] for people. As in this way perhaps you might say: the highly literate, so highly educated and smart people, can do it in this way. Which allows us to pay more attention to those who find it more difficult. Because these [highly literate] people disappear from the waiting list, so to say, which is a welcome development. (Q1C3; HCP 12)

Participants across all groups agreed that digital counseling pathways are potentially less suitable for elderly with limited digital access and literacy (Q1C4). Most participants felt that digital pathways would be most suitable for “younger” relatives (i.e., those between adolescence and 50 years). “I'm not really familiar with all of that digital stuff. But for the youth, it is just self‐evident. I'd rather hold a letter in my hands.” (Q1C4; ARR 7).

Several HCP emphasized that intuitive user design and user involvement are essential prerequisites for developing accessible digital genetic services. Participants agreed that providers of digital tools should actively recognize low digital or health literacy and offer adequate support or alternative services.

### 3.4 Theme 2—Impact on family communication

#### 3.4.1 Supporting probands in informing their relatives

ARR and probands both argued that current family communication in cascade testing relies heavily on the knowledge and efforts of probands. Many probands felt not equipped to address difficult questions from ARR while feeling responsible to inform them correctly and consistently (Q2A1).

I don't know to what extent you influence their [ARR's] choice by being too casual, or maybe too weighty or whatever. … You respond differently to everyone, and I think that it would be of great value if on a digital platform someone neutral with scientific knowledge could inform them about it. (Q2A1; Proband 9)

One proband noted that ARR might feel hesitant to contact or ‘bother’ the proband for further information in an already emotionally stressful time. These participants agreed that digital tools could lower the burden on probands, as ARR would have direct access to relevant information (Q2A2).

It would be nice if you could do it digitally … I think it's a really good idea. Because it's quite difficult otherwise. I had to send everyone letters and emails and this way you could maybe inform the whole family at once. (Q2A2; Proband 3)

Furthermore, participants argued that digital tools could support the cascade process in situations with complex family relations, limited contact, or relatives living abroad. It might lower the threshold to reach out to ARR, either directly or through an HCP. However, HCP also expressed concerns that digital tools might limit options for HCP to intervene or support families with complex relations (Q2A3) due to limited involvement.

For example, uncle is a carrier. Father does not want to perform [predictive] testing, his son does want to test. … So there is a conflict of interest. And then the son could just apply for genetic testing on a website or in an app, while refusing to advocate the father's interest. (Q2A3; HCP 5)

#### 3.4.2 Insights into the cascade process

HCP believed digital tools could enhance insights into who is informed and engaged in cascade testing. However, they also considered it potentially challenging to integrate tools with electronic health records and to remain updated on the cascade testing process within a family (Q2B1).

But also, just for keeping track and having an overview within the family, because I'm afraid you'd lose that otherwise. Who has been there [for predictive counseling]? What are the results? Does everyone who needs to know actually know? (Q2B1; HCP 10)

Several HCP consider pedigrees as a relevant functionality to gain insight into family relations and inheritance. Similarly, some ARR believed pedigrees could also provide insights into carrier status and might motivate other ARR to perform cardiac screening or genetic testing.

### 3.5 Theme 3—Impact on decision‐making

#### 3.5.1 Tailored information in digital counseling tools

Many HCP emphasized the importance of tailoring digital pretest counseling information to counselees' life stage (Q3A1), personal values, and health literacy.

That people understand the pros and cons of a pre‐symptomatic test well… that is very much individually determined. One person might be considering having children or is about to buy a house, perhaps even just about to sign a contract, so to speak. These are all matters that, yes, are addressed on a per‐person basis in counseling. Whether, if you create a kind of generic program, can this indeed adequately filter these out. (Q3A1; HCP 2)

Most HCP expected this to be challenging in a digital context, while ARR and other HCP argued digital tools could improve information provision. For example, online information can be reread, unlike in‐person conversations. Also, digital tools enable information transfer through various formats, such as text, videos, animations, or illustrations. Most probands and ARR emphasized that clear, concise, and basic information is essential for pretest information provision, which was often not experienced during in‐person consultations (Q3A2).

Because I think it's important, very simple language… My tip would be to have it checked by medical professionals to ensure it's okay, but not to let them write it themselves. I really notice that medical terms are thrown around, and you end up feeling like you need to bring a dictionary to your next appointment so I might follow along with some parts. (Q3A2; Proband 7)

Other probands with high educational levels suggested a more layered approach, where complex information is available on demand (Q3A3).

Ideally, you would want a website where the information is explained both simply and scientifically, on two different levels. Right now, they stick to the lowest level, which makes me lose interest. I end up thinking: this is so childish, never mind. (Q3A3; Proband 5)

#### 3.5.2 Trustworthiness of information

ARR and probands considered a digital tool for informing ARR as more objective than information shared within a family, noting that family dynamics can influence how information is provided (Q3B1). “I think using such a portal could actually help to objectify things, so to speak. Because in family relations, it's just never objective. There are always various factors at play.” (Q3B1; ARR 7).

Also, several ARR attempted to gather information through web search engines, which they often found frightening and confusing. In contrast, digital tools, hosted by established medical institutions, would be considered trustworthy and could assist ARR in finding relevant information for their families (Q3B2). “Nowadays, you can Google and find the information, of course. But I would appreciate having all that information gathered [on a website]. Especially from a hospital, which is also trustworthy.” (Q3B2; ARR 9). Participants across all groups agreed that digital counseling content should be reviewed by medical professionals.

#### 3.5.3 (Non)coercive factors

One proband thought that digital pathways were less coercive toward genetic counseling or testing, since no HCP is present to advocate for a test (Q3C1).

I think that when people have that conversation in person, … I believe you're less likely to say no compared to when you're just behind a screen and you say, well, thanks for the information, and I'm closing the conversation now. So I think there's a nuanced difference there. (Q3Q1; Proband 11)

However, some ARR worried that a streamlined digital pathway might nudge an ARR toward quick decision‐making and that this should actively be prevented (Q3C2).

So, if you have such a digital tool, it should also be very clearly stated that: This is not to make a decision for you, but to make you think about it. It's important to emphasize that clearly. … so it doesn't become something coercive. It shouldn't be like: Well, you've read this, so now [you decide]! No. (Q3C2; ARR 13)

Several ARR explained that they needed time, ranging from days to weeks, to think, reflect, and discuss their decision. They argued that digital tools should explicitly allow time for reflection before consenting (Q3C3).

And I also took a few weeks, maybe two or three, to make that decision [about predictive testing]. I didn't make it when I read a letter or browsed the internet. It was ultimately in conversation with my wife or possibly with someone at work, a colleague, that I discussed it. … So, I'm not sure if you should try to enforce that someone makes the decision in that moment on a digital portal. (Q3C3; ARR 6)

Some ARR also believed that pursuing genetic testing is important, and felt that a digital platform should explicitly encourage ARR to undergo genetic testing or cardiac screening, or persuade those in doubt to do so. Additionally, some HCP emphasized that in a digital context they would have less insight into potential coercive influences within the family, such as relatives pressuring someone to make a specific decision, taking over the digital pathway, or to click for someone else (Q3C4). Ideally, these HCP wanted to filter out such cases but questioned the feasibility of doing so. “I think if people want to cause harm, they can do it more easily through a digital environment, so maybe you can't completely prevent that [coersive factors in a family].” (Q3C4; HCP2).

#### 3.5.4 Quality of decision‐making

Many HCP also shared concerns about the impact of digital tools on the quality of informed consent for genetic testing, especially without their involvement (Q3D2). “You should be careful that people might say or think that they understand all consequences, but then in the end, they are worse off. How will you check that?” (Q3D2; HCP 14). Several participants, both HCP and probands, worried that digital pathways might oversimplify the decision‐making process, reducing it to merely ticking a box. This was illustrated by the metaphor of an online webshop transaction, where ARR would opt for genetic testing without careful consideration (Q3D4).

…it's a bit like a webshop. Just like, let's do a quick DNA‐test. On the one hand, this sounds very simple, to find out whether you have it [positive carrier status] in a fast and efficient way. But it should be much more carefully organized than that. (Q3D4; Proband 9)

Most HCP believed that incorporating an in‐person contact opportunity alongside a digital pathway is necessary to ensure ARR's understanding. Some ARR and probands shared this view. In contrast, other HCP argued that for digitally skilled or already well‐informed counselees, additional in‐person counseling would not be required to obtain fully informed consent.

Several HCP stated that digital pathways might empower ARR in their decision‐making on predictive genetic testing, thereby promoting counselee‐centred care. One HCP also stresses the return of result process as an important phase of the counseling process where options to receive results digitally might improve a counselee's autonomy as this might give more room for reflection and formulating questions. Based on their experience with current pathways, they found that counselees are often well‐equipped to decide whether they prefer in‐person, telephone, or video consultations (Q3D1).

In my case, almost 90 percent opt for a phone appointment, so they simply don't want that much contact; they don't necessarily want to see someone in person. … Those who really want a physical appointment and feel a need for it make that clear. So, I think those choices are usually quite well made by the person seeking advice. (Q3D1; HCP 10)

Digital pathways could serve as an additional option within genetic care if counselees always have the choice to opt‐out of digital pathways.

### 3.6 Theme 4—Impact on care relations

#### 3.6.1 Human contact and emotional support

Participants highly valued direct human contact between counselees and HCP in the current care. Some expressed concerns that further digitization could dehumanize genetic care (Q4A1). “I struggle with dehumanization… But that really bothers me, and I don't consider myself a social worker who's overly old‐school, but I do value human contact.” (Q4A1; HCP 15). In‐person contact, either in‐person or via video/telephone, was often described as irreplaceable, especially for decision‐making and psychosocial support (Q4A2).

You can facilitate it [digital consent about genetic testing], but I don't know if it's sensible. I don't know if you should leave it up to people themselves to make such important choices based on online information. I think that it's, I remember from my intake I was asked many questions which made me rethink: do I want this? What are the consequences? What could it all entail? What does it mean when you know {your carrier status]? Or when you don't know? So I think it is wise to talk to someone about it once. (Q4A2; Proband 11)

While direct human contact was not deemed essential for providing standard information or collecting medical and family history, it was considered crucial for adequately interpreting genetic results by both HCP and patients and for clinical follow‐up by HCP (Q4A3).

[in the scenario of full digital consent] … you have no contact at all where you can sense whether this person can process and understand the information well. And indeed will not panic immediately when applying for all kinds of things online and receiving digital results, which this person cannot deal with. That's a whole lot for a robot or computer to assess. (Q4A3; HCP 14).

Many ARRs emphasized that digital counseling tools should always allow users to share concerns or pressing questions with an HCP and that the threshold to do so should be as low as possible. Additionally, a few HCP highlighted the importance of building a care relation during pretest consultation to prepare for the potential return of positive test results. Several HCP noted that digital tools could allow for more meaningful contact for those requiring additional attention. Several participants believed that for many ARR digital counseling might be a valuable contribution, under the condition that in‐person counseling remains an option. Few HCP perceived mandatory in‐person contact moments as paternalistic.

#### 3.6.2 The role of conversational agents in genetic counseling

Several ARR and probands argued that digital alternatives for in‐person contact, such as CA or direct chat functions with HCP, would not be adequate substitutes (Q4B1). “[HCPs] provide more nuance, empathy, just human contact.” (Q4B1; Proband 4). Participants also believed that developers should be cautious when developing CA for psychosocial support. Several HCP emphasized that only HCPs can provide the necessary empathy and nuance (Q4B2).

You just know that you are interacting with a robot, so empathic responses are worth, well nothing. … I'd rather speak to someone face to face or hear a human voice of a healthcare provider that I really need in that moment. (Q4B2; HCP 5)

Some ARR and probands expressed agitation and felt upset when visualizing ‘empathetic’ responses of CA (Q4B3).

You'll feel something like: Oh dear, I might have a terrible disease, what will happen to me? Will I survive? Just a very primary thought. And if you would get a chatbot suddenly. Yes, that would be the biggest anticlimax I can imagine. Because, I would be like, have they just lost their mind at the hospital? (Q4B3; Proband 7)

Some of them even expected counselees to disengage from digital counseling if confronted with a CA that fails to provide satisfactory answers or comes across as overly empathetic. Less emotionally charged topics or information were considered suitable for being transferred via CA if well‐tailored to end‐users. Some HCP stated they were unaware of technological capabilities of CA in the (near) future and whether they could provide thoughtful or empathetic answers. If so, their stance on implementing CA might change.

### 3.7 Theme 5—Impact on the genetic care system

#### 3.7.1 Changes in the profession of genetic counselors

Some HCP expressed concerns that transferring counseling tasks to digital tools could alter the profession of HCP in clinical genetic care. Several HCP described a potential shift in professional identity; in what it means to be a genetic HCP. One HCP worried that the existence of digital counseling tools might question the added value of HCP in genetic care, especially in the absence of human contact (Q5A1). “If we will make these things [digital counseling tools] accessible, that does say something about well, do we [as genetic HCP] add something to a counseling? And I think we sure do.” (Q5A1; HCP 12). Additionally, several HCP felt that digital counseling tools could diminish their job satisfaction.

#### 3.7.2 Responsibility and accountability for digital counseling tools

To ensure the quality of a (partial) digital genetic care pathway, probands and ARR agreed that HCP must be responsible for both the quality of the information that is provided and the individual counseling process (Q5B1).

A [digital] process should be very well and very carefully designed. So, I think it's the responsibility of the HCP, because they're responsible in the end to construct a [genetic care] journey, also on a website. (Q5B1; ARR 9)

Therefore, HCP should be closely involved in the design and development of digital counseling tools. While many HCP underlined these considerations, some argued that in digital pathways without HCP involvement, the responsibility should rest with the clinical center providing the digital genetic service. If individual HCP are responsible for fully digital counseling pathways, they should have control over the information provided, especially when genetic test results are returned digitally. Also, it was argued that HCP should be linked to individual counselees to answer questions accurately, and ensure procedural quality. A few HCP highlighted the responsibilities of technical developers in maintaining quality of online genetic care pathways. HCP argued that implementing digital tools would delegate tasks to ARR of identifying misunderstandings or concerns and to reach out to HCP accordingly. While many HCP questioned whether it is appropriate to place these responsibilities on counselees, some felt it would be appropriate for most ARR.

#### 3.7.3 Efficiency

All participants perceived absence of travel times as an advantage of digital tools. Some probands noted that digital methods to inform ARR could save time compared to in‐person conversations, especially with more distant ARR. Most HCP anticipated that digital tools would drastically reduce counseling time, allowing room for more complex consultations or tasks (Q5C1).

[Online counseling could impact] the full plates of genetic counselors, who could use their time for more useful tasks. Well, “more useful tasks” is such a value judgment, right, but at least tasks where their expertise can flourish better. (Q5C1; HCP1)

One HCP was more skeptical, arguing that predictive counseling consultations are already relatively short and administrative tasks demand a lot of time. Several HCP expected digital tools to reduce waiting lists for genetic counseling. Cost impact was mentioned as another, albeit less emphasized, opportunity: some ARR and HCP expected lower healthcare costs, compared to traditional counseling.

#### 3.7.4 Privacy and digital safety

To ensure responsible implementation, all participant groups flagged the importance of digital safety and privacy. Not only to promote trust but also to safeguard medical and genetic information. Several ARR stressed that insurance companies should not have access to digital cascade testing tools (Q5D1).

Obviously, privacy should be protected. Because that's a threat these days. Well, you never know what people will do with your data. Whether they will use it against you, for example if a mortgage lender or life insurer will search on such a portal. These are things that should be safeguarded, so it's not traceable to individuals. (Q5D1; ARR 1)

Several participants expressed hesitance about using digital tools due to privacy concerns. To address these, secure login methods were suggested along with protecting the confidentiality of personal information. Furthermore, data should not be shared within families without explicit consent.

## 4 DISCUSSION

This focus‐group study revealed a predominantly positive attitude among probands, ARR, and HCP toward digitization of certain aspects of the cascade testing journey including predictive counseling. Expected benefits include saving in‐person counseling time while increasing time for ARR to engage with relevant information, improving information provision at home, and increased accessibility of genetic counseling. However, specific concerns were raised on the applicability of CA for emotional support and the lack of in‐person contact when implementing a fully digitized cascade testing pathway. Furthermore, to promote equitable genetic care, digital genetic testing tools should accommodate counselees with varying levels of (digital) literacy. This aligns with a recent study where HCP emphasized the need to prevent digital tools from further increasing existing inequalities in access to genetic care (Lee et al., 2024). Accessibility for digitally illiterate counselees was already identified as one of the main challenges of implementing BRCA‐DIRECT, a digital germline genetic testing pathway for breast cancer patients (Torr et al., 2022). Additionally, multilingual availability of these tools is essential to ensure equitable access to genetic services for those who do not speak the default language (Cragun et al., 2023). Our findings suggest that providers of digital tools have an active duty to recognize digitally or health‐illiterate counselees and offer support or alternative services. It is argued that both policy and research agendas should prioritize data and digital literacy, as well as language that is used in digital genetic services (Bombard et al., 2022). In other fields, it is suggested that counselees with low digital literacy should be part of co‐creation processes to achieve this (Oldhoff‐Nuijsink et al., 2024).

Participants in this study noted that digital tools can both support and challenge sound decision‐making. Safeguarding access to human contact during digital counseling was the key requirement for clinical integration, also referred to as a “safety net” in a similar study about the acceptability of chatbots in genetic services (Luca et al., 2023). Without in‐person HCP involvement, participants worried that fully digital counseling methods would not ensure personalized decision‐making. Most participants agreed that, in the context of predictive genetic counseling within digital cascade genetic services, hybrid models of care (combining digital and in‐person contact) are the most suitable. Shickh et al. already showed that in‐person consultations following digital pretest counseling enhanced personalized decision‐making aligning with personal values and informed dialogue, compared to in‐person counseling alone (Shickh et al., 2021). This substantiates that digital tools can benefit decision‐making. In contrast, digital tools can also challenge well‐informed decision‐making in a genetic counseling process (van Lingen et al., 2025). Further research is needed to assess potential decisional conflict in counselees, and to better understand the impact of fully digitized or hybrid predictive counseling pathways on decision‐making and quality of informed consent.

There is limited knowledge about which parts of the genetic care pathway are suitable for digitization. Recently, Lee et al. found that HCP were more positive about adopting digital tools in the pretest rather than the posttest phase (Lee et al., 2024). This partly aligns with our findings, though our participants, including probands and ARR, were more skeptical about fully digitizing pretest counseling and decision‐making for predictive testing. Similarly, HCP in Lee et al.'s study favored hybrid models (part in‐person, part digital counseling) for complex cases, while digital tools alone were deemed sufficient for less complex cases (Lee et al., 2024). However, most participants in our study considered a “human in the loop” necessary in all cases. This contrast may be attributed to participants' tendency of hesitance toward digitization, whereas Lee et al.'s cohort was described as prone toward digitization (Lee et al., 2024). Also, the age of ARR in our study was relatively high. All participants expected young adult ARR to be even more positive about digital tools. A chatbot evaluation study by Schmidlen et al. underlined this view in relatives (Schmidlen et al., 2022). Additionally, our findings contribute to the debate on which hereditary conditions and genetic services are suitable for digital interventions, with relevance beyond cardiogenetics. Considerations included disease severity, treatability, and actionability, and psychosocial impact. Participants identified both opportunities and challenges for implementing digital tools in diagnostic mainstreaming settings and in predictive genetic testing of ARRs. Based on these findings, it could be suggested that fields with similar profiles such as hereditary cancer are particularly suitable. Further research is needed to determine which tasks and roles digital tools should adopt within the genetic care process, in which contexts, and for which counselees. We believe that qualitative study designs in particular are most suitable to address this issue. Participants' critical attitude toward CA for psychosocial support aligns with a study by Luca et al. (2023) where HCP considered complex tasks like explaining results or providing emotional support as unsuitable for CA. Instead, implementing digital tools may allow HCP to have more meaningful interactions with other counselees. An evaluation study of the Rosa chatbot, which provides factual information about BRCA testing to breast‐ and ovarian cancer patients alongside genetic counseling, found high patient satisfaction and trust (Siglen et al., 2023). However, participants did miss eye contact and human empathy while using the chatbot (Siglen et al., 2023, p. 6). Both studies emphasize that CA in genetic services should augment rather than fully replace in‐person contact (Luca et al., 2023; Siglen et al., 2023).

Strengths of our study include in‐depth perspectives from representative groups involved in cardiogenetics. Incorporating views from probands, ARR, and HCP offers unique insights into the perspectives and preferences within these groups. Although the focus is on cardiogenetic counseling, the themes and subthemes described are broadly applicable to other diseases and areas within (predictive) genetic counseling. A potential limitation of this study is selection bias of those supportive of genetic testing, higher age, and of participants with adequate health literacy. First, ARR who did not receive counseling were unfortunately not represented, resulting in an underrepresentation of perspectives of those hesitant about genetic testing. Furthermore, all participants expressed support for genetic testing, which might have influenced the perspectives shared as they were motivated to support methods that might increase cascade testing uptake. Second, participants of this study were middle‐aged and beyond with a median age of 54 years for probands and 61 years for ARRs, which does not reflect the mean age of probands (43.8 years; SD 14.6) and ARRs (43.9 years; SD 19.3) dealing with inherited cardiac conditions in the Netherlands (Van Den Heuvel et al., 2020). One explanatory factor could be that children were excluded from this study. Nevertheless, the experiences described in this study may not fully reflect those of the broader target population for digital interventions, which might also include adolescents and young adults who are, generally, more accustomed to and interested in digital approaches. Third, participants in this study reported medium or high health literacy, whereas the prevalence of low (8%) or limited (27%) health literacy combined is around 35% in the Dutch adult population (Heijmans et al., 2024). Because limited health literacy is a well‐known barrier to accessing genetic care (van der Giessen et al., 2021), our findings might lack perspectives on how digital tools might either enhance or limit accessibility of genetic services for counselees, especially for those with low health literacy skills.

In conclusion, digital tools for cardiogenetic counseling are promising in supporting both counselees and HCP in cascade testing. In the clinical setting of predictive counseling, efforts should focus on ensuring access to genetic services for all ARR and protecting quality of care and in‐person involvement of HCP. A majority of our findings extend beyond themes that are exclusively relevant for inherited cardiac conditions, such as digital literacy, human contact, and accessibility. As such, we believe that the results of this study may be applicable to a broader range of (monogenic) genetic conditions that are dealing with cascade genetic testing, including hereditary cancer and nefrogenetics. However, further research is needed to deepen our conceptual and empirical understanding of the opportunities and concerns identified in this study, and to determine their relevance for other genetic conditions and services. Specifically, both qualitative and quantitative research focusing on digital and health literacy, quality of informed consent, the appropriate roles of technology in genetic care, cascade testing uptake, and the role of conversational agents in genetic care warrant additional attention in future research and policy agendas.

## AUTHOR CONTRIBUTIONS

Marlies N. van Lingen, Lieke M. van den Heuvel, Tessa C. Beinema, Randy Klaassen, and Noor A. A. Giesbertz substantially contributed to the study's conceptualization and design. Marlies N. van Lingen prepared materials, recruited participants, and performed focus groups with substantial contributions from all authors. Data acquisition was performed by Marlies N. van Lingen, Lieke M. van den Heuvel, Noor A. A. Giesbertz, and Tessa C. Beinema. Data analysis and interpretation were performed by Marlies N. van Lingen, Sietske A. L. van Till, and Lieke M. van den Heuvel, with substantial input from Martina C. Cornel, Randy Klaassen, Margreet G. E. M. Ausems, and J. Peter van Tintelen. Marlies N. van Lingen drafted the manuscript and designed illustrations. J. Peter van Tintelen and Lieke M. van den Heuvel acquired funding and supervised Marlies N. van Lingen. All authors critically revised the different versions of the manuscript for important intellectual content. All authors gave final approval for this version of the manuscript to be published. Marlies N. van Lingen, Sietske A. L. van Till, and Lieke M. van den Heuvel had full access to all study data and took responsibility for data integrity and the accuracy of the analysis.

## FUNDING INFORMATION

This project was financially supported by ZonMW/IMDI and the Dutch Heart Foundation under Grant numbers 104021006 (ZonMW) and 2019B012 (Dutch Heart Foundation).

## CONFLICT OF INTEREST STATEMENT

M.N. van Lingen, S.A.L. van Till, N.A.A. Giesbertz, T.C. Beinema, M.G.E.M. Ausems, R. Klaassen, M.C. Cornel, L.M. van den Heuvel, and J.P. van Tintelen declare that they have no conflict of interest.

## ETHICS STATEMENT

The study protocol was exempted from approval by the Medical Ethical Committee NedMec because the Act of Medical Research Involving Human Subjects (WMO) was not applicable (research proposal no. 21‐474/C).

Human studies and informed consent: We obtained written and verbal informed consent from all participants in this study. All procedures followed were in accordance with the ethical standards of the responsible committee on research involving humans (institutional and national).

Animal studies: Animal studies were not applicable.

## Supporting information


Appendix S1

Appendix S2

Appendix S3

## Data Availability

Anonymized data are available from the corresponding author upon reasonable request. The codebook is available upon request.
