# Supplementary material for: Digital genetic counseling services for cascade cardiogenetic testing
Source: J Genet Couns. 2026 Apr 29;35:e70208. doi: 10.1002/jgc4.70208 (PMC13127103; doi:10.1002/jgc4.70208)
Supplement: Supplementary file 2 — Appendix S2 [file JGC4-35-0-s001.pdf]

Supplement 2 – Questionnaires to obtain participant characteristics

Questions below were originally discussed in Dutch during the participant interviews and have been translated for publication purposes.

Probands

1. The focus group discussion will take place online via MS Teams. Do you have access to a laptop or tablet, and are you able to use it once we send you an invitation link?
   a. Do you need any assistance with using MS Teams?
   b. Do you give permission for audio and video recording?
2. You have a hereditary heart disease. Which hereditary heart disease has been diagnosed?
   a. In which year was this hereditary heart disease diagnosed?
3. How many family members were you asked to inform about the hereditary heart disease / who are at risk for your hereditary heart disease?
4. What is your family situation?
5. In what ways have you obtained information about the hereditary heart disease?
6. Have you been able to inform your family members?
7. Did you receive assistance from the Department of Clinical Genetics / a clinical geneticist in informing your family members?
8. Did you receive a family letter from the clinical geneticist to share with your relatives?

I will now ask you a few questions about your education and background. I am asking these because we are interested in your perspective on new developments and would also like to know more about the background of the participants.

9. What is the highest level of education you have completed?
10. Country of birth of your parents and yourself
11. How often do you need help reading letters or brochures from your general practitioner, the hospital, or other healthcare institutions? Never, occasionally, often, or always? (health literacy)
12. How often do you need help using your computer, the internet, or functions such as DigiD? Never, occasionally, often, or always? (digital literacy)

At-risk relatives

1. The focus group/discussion will take place online via MS Teams. Are you able to use it once we send you the link?
   a. Do you need any assistance with using MS Teams?
   b. Do you give permission for audio and video recording?
2. You have one or more relatives with a hereditary heart disease. Which disease is it?
   a. Which variant?
   b. How are you related to the family member with the hereditary heart disease?
3. What is your family situation?
4. When were you informed about the heart disease?
   a. Who informed you?
   b. Did you receive the family letter at that time?
5. How did you obtain information about the heart disease?
6. What was your decision regarding predictive genetic testing?

I will now ask you a few questions about your education and background. I am asking these because we are interested in your perspective on new developments and would also like to know more about the background of the participants.

7. What is the highest level of education you have completed?
8. Country of birth of your parents and yourself
9. How often do you need help reading letters or brochures from your general practitioner, the hospital, or other healthcare institutions? Never, occasionally, often, or always? (health literacy)
10. How often do you need help using your computer, the internet, or functions such as DigiD? Never, occasionally, often, or always? (digital literacy)

Genetic Healthcare Professionals

1. May I ask your gender?
2. What is your age?
3. What is your position or role within the hospital?
4. How long have you been working in this role?
5. In which hospital do you work?
