# Supplementary material for: Digital genetic counseling services for cascade cardiogenetic testing
Source: J Genet Couns. 2026 Apr 29;35:e70208. doi: 10.1002/jgc4.70208 (PMC13127103; doi:10.1002/jgc4.70208)
Supplement: Supplementary file 3 — Appendix S3 [file JGC4-35-0-s003.pdf]

Supplement 3 – Topic list focus group discussions

Topics and questions below were originally discussed in Dutch during the focus group discussions and have been translated for publication purposes. The questions were used as a guide during discussions. Follow-up subquestions were only asked by the moderator if necessary to enhance discussion about a particular topic.

Part 1: The concept of a digital clinic

Discussed during focus group sessions with the following participant groups: probands, at-risk relatives (ARR), healthcare professionals (HCP)

THE DIGITAL CLINIC – CONCEPT

Imagine there were a secure “digital clinic” for your family members, in the form of a website or portal (these terms refer to the same thing: an online platform for families with a hereditary heart condition). Within the digital clinic, family members could find information about the hereditary heart condition and genetic (DNA) testing. If desired, they could also request anat-home genetic test.

[what comes to mind]

1) When I mention a digital clinic, what comes to mind?
   a) What should a clinic be able to do, and what should it look like?
      i) Do you think a website or rather an app would be more suitable for this?

[concept]

2) What do you think about the idea of a digital clinic for genetic counseling?

[advantages / disadvantages]

3) What advantages do you see in such a digital clinic? And what disadvantages?

[telling relatives about the clinic]

4) Suppose such a digital clinic had existed when the hereditary heart condition was first diagnosed in your family—would you have told your relatives about it?
   a) Do you think relatives would make use of such a digital clinic? Could you elaborate?
   b) Do you think such a digital clinic could be suitable for all relatives? Could you elaborate? For whom would it be suitable, and for whom not?

[privacy]

5) What are your thoughts on privacy in relation to such a digital clinic?
   a) Why is it (not) important?
   b) How should this be handled?

Part 2 – Informing probands about the digital clinic

Discussed during focus group sessions with the following participant groups: HCPs only

INFORMING THE PROBAND ABOUT A DIGITAL CLINIC

A digital clinic consists of several steps. We would like to ask you to think along with us. First, let us consider how a proband should inform relatives about the hereditary heart condition while using a digital tool.

[timing of discussing the clinic]

1) At what point in the genetic care journey should the digital clinic be discussed with probands?

[proband choice to use or not use the clinic]

2) Should probands be allowed to decide whether their relatives can or cannot use the digital clinic? Could you elaborate?

a) If probands use a digital clinic, should they be able to indicate which relatives they consider (un)suitable for it?

[suitable user group]

3) Do you consider relatives eligible for presymptomatic DNA testing an appropriate target group for a digital care pathway?

Part 3 – Informing relatives

Discussed during focus group sessions with the following participant groups: probands, ARR, HCP

INFORMING RELATIVES ABOUT THE DIGITAL CLINIC + LOGIN

Relatives with a hereditary heart condition in the family could make use of a fictive digital clinic.

[informing relatives about the existence of the clinic]

1) How should relatives be informed about the hereditary heart condition in the family and the existence of the digital clinic?

a) Who should inform them about these matters? (For instance: proband, another family member, or a healthcare professional from the Clinical Genetics department? What do you envision?)

b) Through which medium (e.g., email, WhatsApp/SMS, letter) should this be done?

[how relatives access the clinic]

2) If a relative is interested, how should they gain access to the clinic?

a) For example, via the hospital patient portal, email, letter, or link provided by the relative with the hereditary heart condition.

[personal login]

3) Do you think each relative should have a personal account or login environment in the clinic?

a) What would that look like in your opinion?

Part 4 – Information provision

Discussed during focus group sessions with the following participant groups: probands, ARR, HCP

INFORMATION PROVISION

We are curious about your perspective on how information could best be presented in a digital clinic.

[what information]

1) What type of information should be provided in the digital clinic?

[how information is presented]

2) In what way should the information be provided?
   a) Which format/medium (e.g., text, video, etc.)
   b) Should all information be given at once, or step by step? Why do you think so?

[possible contact with healthcare provider]

3) In principle, someone could use the digital clinic without direct contact with a healthcare provider. How do you envision possible contact with a Clinical Genetics professional or social worker/psychologist?
   a) Should they provide support when needed, or more structurally? At which moments?

[personal decision]

4) Do you think the clinic could support a personal decision about whether or not to undergo predictive genetic testing?
   a) In the current situation, people discuss with a healthcare provider whether they want to/ whether it is the right time to undergo predictive genetic testing. Do you think a digital clinic could also support this process?
   b) Do you think a human or technical check is needed to ensure that someone has completed all steps and truly understands the information?

[own choice / no pressure]

5) It is important that individuals make their own decision about undergoing DNA testing and do not feel pressured by others. If this decision were made digitally, how do you envision that?

[responsibility]

6) Currently, a healthcare provider in Clinical Genetics is responsible for ensuring proper care for at-risk relatives. Who do you think should be responsible in the case of a digital clinic?

Part 5 – Chatbot or virtual assistant

Discussed during focus group sessions with the following participant groups: probands, ARR, HCP

The term "chatbot" used below was replaced with "virtual assistant" after the first round of focus group discussions with each subgroup.

CHATBOT / VIRTUAL ASSISTANT

The platform may also include a chatbot or virtual assistant—a computer-based conversational partner that can assist users in various ways.

[what comes to mind]

1) What comes to mind when you hear the term chatbot/virtual assistant?

A chatbot/virtual assistant can be designed in different ways. For instance: in example A, the entire conversation with the chatbot/virtual assistant contains all the information; in example B, the collaboration between the chatbot/virtual assistant and the digital clinic content is clearly visible; or in example C, the information and conversation are presented in a more playful, interactive way.

A: 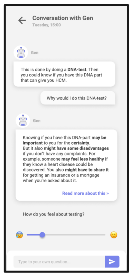

B: 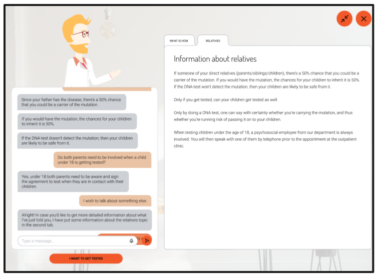

C: 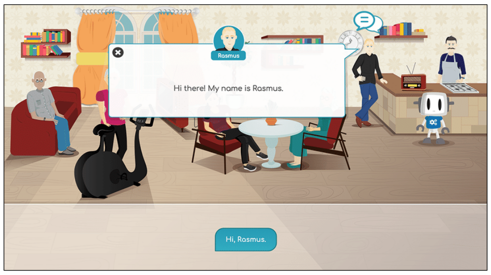

(slide overview shown) These are some examples to give you an idea of how a chatbot/virtual assistant could appear in a digital clinic.

[your opinion]

2) What do you think about the idea of a chatbot/virtual assistant?

[ways to provide information]

3) The chatbot/virtual assistant could help people obtain information in several ways. What would be the best approach?

a) For example, it could answer questions directly, or it could gradually reveal information—allowing users to navigate the clinic together with the chatbot/virtual assistant. What are your thoughts?

b) Would you prefer one approach over the other? Why?

[emotional support]

4) The chatbot/virtual assistant could also be designed to respond empathetically or ask about users' feelings regarding certain topics. How would you feel about that?

a) In what way would you prefer the chatbot/virtual assistant to do so?

i) Suggestions: Only in text? With facial expressions? Exaggerated or subtle?

[considerations for design]

5) What else do you consider important for us to take into account when designing the chatbot/virtual assistant?

a) For example, should it reach out proactively or only respond when users ask something? Should it remain professional or appear more informal/personal?

Part 6 – Decision-making about genetic testing

Discussed during focus group sessions with the following participant groups: probands, ARR, HCP

DECISION ABOUT GENETIC TESTING

Suppose a relative decides to undergo predictive genetic testing.

[indicating willingness for a genetic test]

1) How should a relative indicate that they wish to undergo a genetic test?
   a) Could/should this be done through the clinic, or should there still be contact with a Clinical Genetics healthcare provider?

[home genetic test]

2) What are your thoughts on a home-based genetic test (where DNA is collected at home using a cheek swab)? Could you elaborate?

[receiving the genetic test results]

3) How should the genetic test results be provided to relatives?
   a) Should this be done through the digital clinic, or would you prefer a letter or personal contact with the healthcare provider?

Suppose a relative decides not to undergo genetic testing at this time.

[indicating non-participation]

4) How should a relative indicate that decision?
   a) Could/should this be done through the clinic, or should there still be contact with the Clinical Genetics healthcare provider?

[availability of the clinic]

5) Do you think the clinic should remain available for relatives, so that they can reconsider at a later moment?

[referral for cardiological screening] (HCP only)

6) How do you envision referrals for cardiological screening within a digital clinic?
   a) Should relatives be asked within the digital clinic whether they wish to be referred for cardiological screening? Why or why not?

Part 7 – End of focus group

CLOSING

1) Are there any additions or questions regarding the topics we discussed?
